# Vitamin B deficiency and sarcopenia: an integrated narrative review of metabolic, inflammatory, endoplasmic reticulum stress, and myokine signaling pathways

**DOI:** 10.12701/jyms.2026.43.51

**Published:** 2026-08-03

**Authors:** Kisang Kwon, Sangmin Lee, Jenny Kwon, Seung Whan Kim

**Affiliations:** 1 Department of Biomedical Laboratory Science, Wonkwang University, Iksan, Korea; 2 Department of Bio-Environmental Chemistry, College of Agriculture & Life Sciences, Chungnam National University, Daejeon, Korea; 3 Philip R. Lee Institute for Health Policy Studies, University of California, San Francisco, CA, USA; 4 Department of Epidemiology & Biostatistics, University of California, San Francisco, CA, USA; 5 Department of Emergency Medicine, Chungnam National University College of Medicine, Daejeon, Korea

**Keywords:** Muscle protein metabolism, Myokines, Older adults, Sarcopenia, Vitamin B complex

## Abstract

Sarcopenia is an age-related skeletal muscle disorder characterized by progressive decline in muscle mass, strength, and physical performance, leading to frailty, disability, falls, and increased mortality. Although its pathogenesis is multifactorial, growing evidence indicates that vitamin B complex deficiency contributes to muscle deterioration through interconnected metabolic and signaling pathways. This narrative review summarizes current evidence regarding the roles of B vitamins in skeletal muscle biology and their potential contribution to sarcopenia. Vitamin B deficiency impairs mitochondrial energy metabolism by reducing cofactor availability and adenosine triphosphate production, thereby increasing oxidative stress and chronic inflammation. These disturbances may trigger endoplasmic reticulum stress and the integrated stress response, leading to activating transcription factor 4-dependent induction of growth differentiation factor 15 and fibroblast growth factor 21 expression. Collectively, these changes disrupt protein homeostasis, suppress anabolic signaling, impair neuromuscular function, and alter myokine secretion by reducing anabolic mediators while increasing catabolic and inflammatory myokines, thereby accelerating muscle loss and functional decline. Current evidence is the strongest for vitamins B6, B9, and B12, whereas mechanistic and clinical data for B2, B3, B5, and B7 remain limited. Overall, vitamin B deficiency can be viewed as a modifiable biological contributor to sarcopenia. Well-designed prospective studies and randomized clinical trials are required to clarify causality, validate biomarkers, and determine whether targeted vitamin B supplementation can enhance exercise- and nutrition-based strategies for preserving muscle health in older adults.

## Introduction

Population aging has become a major global demographic trend, with the proportion of older adults rapidly increasing in developed and developing countries. As life expectancy continues to increase, preserving functional independence and promoting healthy aging have become major public health priorities [1]. Among age-related disorders, sarcopenia has emerged as a leading cause of frailty, disability, falls, hospitalization, and mortality, imposing an increasing burden on healthcare and long-term care systems worldwide [2,3]. South Korea, one of the fastest aging countries among the OECD (Organization for Economic Co-operation and Development) countries, officially became a super-aged society in December 2024, highlighting the growing public health importance of effective strategies to prevent age-related muscle decline [4].

Skeletal muscles account for approximately 40% of total body weight and are essential for locomotion, energy metabolism, and endocrine signaling [5]. Age-related muscle loss accelerates after the sixth decade of life, resulting in a progressive decline in muscle mass, strength, and physical performance [2]. Reflecting its growing clinical importance, sarcopenia is now recognized as a distinct disease entity with an International Statistical Classification of Diseases and Related Health Problems, 10th Revision (ICD-10) code and is diagnosed according to the criteria proposed by the European Working Group on Sarcopenia in Older People (EWGSOP), International Working Group on Sarcopenia, and Asian Working Group for Sarcopenia (AWGS) [6-8]. Recent advances in molecular biology and multi-omics have further expanded the identification of biomarkers related to muscle metabolism, inflammation, and nutritional status, thereby providing new insights into the biological mechanisms underlying sarcopenia [9,10].

The development of sarcopenia is multifactorial and involves aging, physical inactivity, chronic diseases, hormonal alterations, mitochondrial dysfunction, chronic inflammation, nutritional imbalance, and genetic susceptibility [3,7,11]. Among these factors, nutritional status is particularly attractive because it is potentially modifiable. In particular, the vitamin B complex has received increasing attention because its members serve as essential cofactors for mitochondrial adenosine triphosphate (ATP) production, amino acid and one-carbon metabolism, antioxidant defense, DNA synthesis, and neuromuscular function [12-14]. Vitamin B deficiency is common in older adults, especially among long-term care residents, owing to inadequate dietary intake, dysphagia, malabsorption, chronic diseases, and polypharmacy [15-17]. Additionally, the increasing use of glucagon-like peptide-1 (GLP-1) receptor agonists has increased the awareness of nutritional monitoring, including protein intake and vitamin B status, as potential modifiable determinants of treatment-associated muscle loss.

Emerging evidence suggests that inadequate vitamin B status contributes to skeletal muscle dysfunction through impaired energy metabolism, oxidative stress, mitochondrial dysfunction, chronic inflammation, neuromuscular impairment, dysregulated protein turnover, and altered myokine signaling [18-20]. Although individual B vitamins participate in distinct biochemical pathways, many of their downstream effects converge on common cellular stress-response mechanisms. Disturbances in amino acid metabolism, redox homeostasis, and mitochondrial bioenergetics may induce endoplasmic reticulum (ER) stress and activation of the integrated stress response (ISR), leading to activating transcription factor 4 (ATF4)-dependent induction of growth differentiation factor 15 (GDF15) and fibroblast growth factor 21 (FGF21) expression. These stress mediators may subsequently influence myokine secretion, muscle protein turnover, mitochondrial adaptation, and regenerative capacity, providing a plausible mechanistic framework linking vitamin B deficiency to sarcopenia [21-23].

However, despite these advances, important gaps remain. Clinical evidence supporting the causal relationships between vitamin B deficiency and sarcopenia is considerably weaker than the mechanistic evidence derived from experimental studies, and the strength of the evidence differs substantially among individual vitamin B subtypes. Furthermore, the molecular pathways connecting vitamin B deficiency to metabolic dysfunction, inflammatory signaling, ER stress, ISRs, and myokine regulation have not been comprehensively synthesized into a unified conceptual framework.

Therefore, this narrative review summarizes the current evidence regarding the mechanistic roles of individual vitamin B subtypes in skeletal muscle biology and integrates metabolic, inflammatory, neuromuscular, ER stress/ISR, GDF15, FGF21, and myokine-mediated pathways into a unified framework to understand sarcopenia. We also identify current evidence gaps and discuss future directions for biomarker development, precision nutrition, and vitamin B-targeted interventions to preserve muscle health and promote healthy aging (Table 1).

The literature was identified through PubMed/MEDLINE using combinations of the terms *sarcopenia*, *vitamin B complex*, *thiamine*, *riboflavin*, *niacin*, *pantothenic acid*, *pyridoxine*, *biotin*, *folate*, *cobalamin*, *myokine*, and *skeletal muscle*. English-language articles published primarily between 2015 and 2025 along with earlier seminal studies were included. Reference lists were manually screened for additional relevant publications. As this was a narrative review, no formal quality assessment or meta-analysis was performed, and the study selection was based on relevance and mechanistic significance.

## Pathophysiology of sarcopenia

Before examining the specific contribution of vitamin B deficiency, this section outlines the clinical burden, current treatment landscape, and molecular pathophysiology of sarcopenia, which provides the backdrop for the vitamin B-centered framework developed in subsequent sections.

### 1. Definition, staging, and clinical burden of sarcopenia

Sarcopenia, first described by Rosenberg in 1997, is an age-related skeletal muscle disease characterized by progressive loss of muscle mass, strength, and physical performance, resulting in frailty, falls, disability, and loss of independence [24]. It is now recognized as a major public health challenge in aging populations. According to the revised EWGSOP (EWGSOP2) and AWGS 2019, the diagnosis is based on three core domains: muscle strength, muscle mass, and physical performance, with low muscle strength considered the primary indicator of probable sarcopenia [25]. Although sarcopenia has historically been classified as presarcopenia, sarcopenia, and severe sarcopenia, the current consensus places greater emphasis on functional impairment than on muscle mass alone [25]. Frailty frequently overlaps with sarcopenia and is commonly assessed using the Fried phenotype, which includes weight loss, exhaustion, reduced physical activity, slow gait speed, and weak grip strength [26]. Data from the Korea National Health and Nutrition Examination Survey demonstrated progressive age-related decline in appendicular skeletal muscle mass and a substantial burden of sarcopenia among older adults, emphasizing the importance of early prevention [27].

Age-related muscle loss preferentially affects type II muscle fibers, leading to a progressive decline in muscle strength and physical performance [28]. Reduced handgrip strength and slower gait speed are key predictors of impaired activities of daily living, falls, and disability [25,29]. Loss of muscle mass also contributes to reduced basal metabolic rate, insulin resistance, metabolic dysfunction, and sarcopenic obesity [30]. Together, these structural and functional changes substantially increase hospitalization, mortality, and healthcare burden in older adults [31].

### 2. Status of sarcopenia treatment development

Sarcopenia is now recognized as a disease associated with falls, fractures, functional decline, and increased mortality rather than an inevitable consequence of aging. Despite growing recognition, management remains centered on non-pharmacological interventions, particularly resistance exercise and nutritional supplementation, because no pharmacological therapy has yet been approved by the U.S. Food and Drug Administration or the European Medicines Agency, and most drug candidates remain in early clinical development [32]. Myonuclear apoptosis contributes to age-related muscle loss, providing a therapeutic target for intervention [32]. Current drug development strategies focus on increasing muscle mass through stimulation of protein synthesis, including myostatin inhibition, insulin-like growth factor-1 (IGF-1) signaling, and testosterone-related pathways, while improving muscle function through G protein-coupled receptor agonists, mitochondrial-targeting agents, neuromuscular junction (NMJ)-stabilizing therapies, and anti-inflammatory or metabolic modulators [7,33-35]. Although the growing clinical burden of sarcopenia has accelerated therapeutic development, no pharmacological treatment has yet achieved regulatory approval, underscoring the continued importance of preventive and nutritional strategies, including the optimization of vitamin B status [36].

### 3. Modifiable and non-modifiable risk factors

Sarcopenia develops through interactions between non-modifiable factors, including aging, hormonal changes, and genetic predisposition, and modifiable factors such as physical inactivity, inadequate nutrition, medication use, and chronic diseases. Among these, regular exercise remains the most effective intervention, with resistance training promoting muscle protein synthesis, aerobic exercise improving endurance, and balance training reducing fall risk [37]. Adequate intake of high-quality protein (1.0–1.2 g/kg/day for healthy older adults) is also essential to counteract age-related anabolic resistance [38]. Smoking cessation, moderation of alcohol intake, maintenance of healthy body weight, and optimal management of chronic diseases further support muscle health [37].

Medication use is an increasingly recognized modifiable contributor to sarcopenia [39]. Long-term glucocorticoids, statins, chemotherapeutic agents, human immunodeficiency virus protease inhibitors, proton pump inhibitors, GLP-1 receptor agonists, and diuretics have all been associated with muscle loss or dysfunction through diverse mechanisms [40-46]. In addition, chronic polypharmacy may impair dietary intake, intestinal absorption, or cellular metabolism of B vitamins, particularly B12 and B6, thereby aggravating mitochondrial dysfunction, neuromuscular impairment, and muscle wasting [47]. Consequently, medication-induced sarcopenia and vitamin B deficiency may act synergistically by overlapping metabolic, mitochondrial, and neuromuscular pathways, further increasing the risk of functional decline in older adults [48].

### 4. Molecular pathophysiology of sarcopenia

Sarcopenia is characterized by an imbalance between reduced anabolic signaling and enhanced catabolic pathways. Age-related suppression of mechanistic target of rapamycin (mTOR) and IGF-1 signaling decreases muscle protein synthesis, whereas activation of the ubiquitin-proteasome system (UPS), principally through atrogin-1 and muscle RING-finger protein-1 (MuRF1), accelerates protein degradation [4,49]. Dysregulated autophagy (LC3, Atg7, and Beclin-1), increased myostatin/activin receptor type-2B signaling, and overexpression of dual-specificity phosphatase 22 further promote muscle wasting [49,50]. Chronic inflammation, particularly via tumor necrosis factor alpha (TNF-α) and interleukin (IL)-6, amplifies these catabolic processes, whereas impaired nuclear factor erythroid 2-related factor 2 (NRF2) signaling increases oxidative stress and frailty [11,51]. Mitochondrial dysfunction and excessive reactive oxygen species (ROS) production are recognized as central contributors to underlying age-related muscle degeneration [20]. Recent artificial intelligence (AI)-assisted analyses, transcriptomic studies, and genome-wide association studies have identified several candidate genes and biomarkers associated with muscle aging, providing new opportunities for precision diagnosis and targeted interventions [52-54].

Collectively, these mechanisms indicate that an inadequate vitamin B status may exacerbate sarcopenia by disrupting mitochondrial bioenergetics, redox homeostasis, protein turnover, inflammatory signaling, neuromuscular function, and cellular stress responses (Table 2). These pathways interact with aging, physical inactivity, chronic diseases, and other nutritional factors, as summarized in Fig. 1.

## Vitamin B deficiency and sarcopenia

The mechanistic contributions of each vitamin B subtype to sarcopenia are summarized in Table 1. Thiamine (B1), riboflavin (B2), niacin (B3), pantothenic acid (B5), pyridoxine (B6), biotin (B7), folate (B9), and cobalamin (B12) are water-soluble cofactors that are indispensable for energy and amino acid metabolism in muscle cells and for muscle protein synthesis and repair. Older adults face elevated deficiency risk due to inadequate intake, absorption difficulties, and reduced appetite. Vitamin B deficiency may impair muscle energy metabolism and protein synthesis while affecting neuromuscular function, thereby potentially contributing to muscle weakness and functional decline, while also inducing neuromuscular conditions such as peripheral neuropathy that indirectly worsen sarcopenia by causing weakness, instability, and altered gait, feeding a cycle of reduced activity and muscle wasting [55]. Maintaining an adequate vitamin B status may represent one component of a comprehensive nutritional strategy for preserving skeletal muscle health, supported by diets rich in whole grains, pork, legumes, and nuts, alongside resistance exercise and healthy lifestyle habits [56].

### 1. Vitamin B1 deficiency

Muscle weakness due to vitamin B1 (thiamine) deficiency arises primarily because of compromised energy metabolism and nerve damage [57]. Thiamine pyrophosphate (TPP) is a cofactor for mitochondrial enzymes, including the pyruvate dehydrogenase and α-ketoglutarate dehydrogenase complexes [58], and TPP deficiency reduces ATP synthesis, raises lactic acid and ROS levels, and triggers muscle cell injury or apoptosis. The resulting energy shortage and elevated ROS levels inhibit mTOR signaling, shifting the balance toward catabolism. Deficiency also impairs neuronal energy availability and the pentose phosphate pathway, disrupting lipid and DNA synthesis in neurons and causing demyelination that disrupts NMJ signaling, contributing to neurogenic muscle atrophy [13,59], of which dry beriberi is a prominent clinical manifestation. Thiamine deficiency additionally provokes low-grade inflammation via IL-6 and TNF-α [57], activating the UPS and autophagy-lysosome pathway to accelerate myofibrillar protein degradation [13,59]. However, it should be noted that the mechanisms described above are predominantly derived from cell-based and animal studies of thiamine deficiency; direct clinical evidence linking vitamin B1 status to sarcopenia outcomes in humans remains limited.

### 2. Vitamin B2 deficiency

Vitamin B2 (riboflavin) functions as flavin adenine dinucleotide (FAD) and flavin mononucleotide, coenzymes essential for oxidation-reduction reactions [60], and its deficiency accelerates muscle dysfunction through three mechanisms. First, FAD is a coenzyme for succinate dehydrogenase (complex II) in the electron transport chain (ETC), and riboflavin deficiency impairs ETC activity and ATP generation, accelerating muscle dysfunction and atrophy [61]. Second, FAD is required by glutathione (GSH) reductase, which regenerates the antioxidant GSH; its deficiency lowers intracellular GSH levels, raising oxidative stress that damages muscle proteins and DNA and triggers inflammation, proteolysis, and apoptosis [60]. Third, riboflavin deficiency impairs disulfide bond formation in the ER, activating the unfolded protein response and disturbing muscle cell homeostasis [22]. Riboflavin deficiency also impairs the NMJ, compounding age-related NMJ degeneration and intensifying atrophy [21]. However, these mechanistic pathways are largely based on cellular and animal models of riboflavin deficiency, and human clinical data directly linking vitamin B2 status to sarcopenia are currently lacking.

### 3. Vitamin B3 deficiency

Vitamin B3 (niacin) is a precursor of the key coenzymes nicotinamide adenine dinucleotide (NAD) and NAD phosphate [18]. NAD serves as an electron acceptor in oxidative phosphorylation; niacin deficiency lowers NAD, reducing ETC efficiency and ATP production [19], thereby impairing muscle strength and performance while raising ROS-driven oxidative stress and nuclear factor kappa B (NF-κB)-mediated atrophy. Because sirtuin 1 (SIRT1), an NAD-dependent deacetylase central to aging and metabolic regulation, requires NAD as a substrate, lowered NAD levels suppress SIRT1 activity, reducing protein kinase B (Akt)/mTOR-mediated growth signaling and forkhead box O-mediated catabolic control, while upregulating MuRF1 and atrogin-1 in the UPS [19]. SIRT1 also activates peroxisome proliferator-activated receptor gamma coactivator 1-alpha, a master regulator of mitochondrial biogenesis; its reduced activity further compromises mitochondrial capacity and muscle energy metabolism [18,19]. Poly(ADP-ribose) polymerase overactivation further depletes intracellular NAD and promotes apoptosis and muscle dysfunction [18]. However, the NAD+/SIRT1-related mechanisms outlined above are supported chiefly by cell and animal studies; direct human evidence connecting niacin (vitamin B3) status with sarcopenia remains sparse.

### 4. Vitamin B5 deficiency

Vitamin B5 (pantothenic acid) is a precursor of coenzyme A (CoA), an essential cofactor for carbohydrate, lipid, and amino acid metabolism. Through its role in acetyl-CoA and succinyl-CoA formation, CoA supports mitochondrial ATP production and skeletal muscle energy homeostasis [62,63]. Accordingly, pantothenic acid deficiency may theoretically impair mitochondrial energy metabolism and muscle function [64]. However, current evidence is derived almost entirely from biochemical principles and preclinical studies and direct evidence linking vitamin B5 deficiency to age-related sarcopenia is lacking, as existing studies have primarily focused on CoA metabolism or inherited metabolic disorders rather than skeletal muscle aging [64,65]. Therefore, any contribution of vitamin B5 deficiency to sarcopenia remains biologically plausible, but speculative, and requires confirmation in mechanistic and prospective clinical studies.

### 5. Vitamin B6 deficiency

Pyridoxal 5′-phosphate (PLP), the active form of vitamin B6, is a coenzyme for over 100 enzymatic reactions, including amino acid metabolism [14], and its deficiency drives sarcopenia through several mechanisms. PLP is required for aminotransferases involved in muscle protein synthesis, and its deficiency impairs amino acid assimilation, favoring protein breakdown over synthesis [14], while also altering Akt/mTOR signaling and activating NF-κB to worsen atrophy [63,66]. PLP is also essential for the methionine cycle and transsulfuration pathway, including cystathionine-β-synthase, which detoxifies homocysteine; PLP deficiency causes hyperhomocysteinemia that contributes directly to atrophy and heightens oxidative stress and inflammation [65]. PLP further serves the kynurenine pathway of tryptophan metabolism, and its deficiency alters neurotoxic and proinflammatory metabolite production, increases muscle inflammation and dysfunction, and enhances NOD-like receptor family pyrin domain containing 3 inflammasome signaling via P2X7 receptors, thereby amplifying inflammatory injury [14,23]. Finally, B6 deficiency impairs muscle satellite cell function and regenerative capacity, hampering repair after injury and substantially contributing to sarcopenia progression [63,66]. However, it is important to note that the proposed mechanisms are supported by a combination of experimental studies, observational human data, and emerging clinical evidence, and causal relationships require confirmation through prospective interventional trials.

### 6. Vitamin B7 deficiency

Vitamin B7 (biotin) serves as a cofactor for several carboxylases involved in glucose, fatty acid, and branched-chain amino acid metabolism, thereby supporting mitochondrial energy metabolism [63,64]. Although biotin deficiency can theoretically impair ATP production and protein metabolism, direct evidence linking impaired biotin status to sarcopenia is currently lacking [63-65]. The existing evidence is limited to biochemical and experimental observations, with no longitudinal or interventional studies demonstrating independent effects on age-related muscle loss. Therefore, the proposed mechanisms should be regarded as hypothesis-generating and further mechanistic and clinical studies are warranted.

### 7. Vitamin B9 deficiency

Vitamin B9 (folate) is active as 5-methyltetrahydrofolate and is essential for one-carbon metabolism [65]. Deficiency impairs the de novo thymidylate synthesis required for DNA replication and repair, hindering the muscle cell proliferation and satellite cell activation required for myofiber repair, and contributing to genomic instability, DNA damage, and cellular senescence, which impair muscle cell function [62,64]. Folate deficiency also impedes Akt/mTOR phosphorylation and downstream ribosomal protein S6 kinase beta-1 and eukaryotic translation initiation factor 4E-binding protein 1 activities, thereby suppressing muscle protein synthesis, differentiation, and myotube formation [64]. As 5-methyltetrahydrofolate works with vitamin B12 to convert homocysteine to methionine, folate deficiency raises homocysteine levels, causing hyperhomocysteinemia that increases ROS levels, impairs mitochondria, promotes apoptosis and inflammation (IL-6, TNF-α), and further suppresses mechanistic target of rapamycin complex 1 (mTORC1) signaling [66]. Homocysteine is also neurotoxic to peripheral nerves, indirectly causing neurogenic atrophy [63]. Reduced synthesis of the methyl donor S-adenosylmethionine further disturbs DNA methylation and induces abnormal expression of DNA damage-inducible transcript 4, an mTORC1 inhibitor, further impeding muscle protein synthesis [64,65]. Multiple observational studies, together with experimental investigations, consistently support the role of folate deficiency in impaired muscle metabolism and myokine regulation; however, definitive evidence from randomized supplementation trials remains limited.

### 8. Vitamin B12 deficiency

Vitamin B12 (cobalamin) is a cofactor for methionine synthase, and its deficiency disrupts homocysteine-to-methionine conversion, causing hyperhomocysteinemia with neurotoxic effects that produce peripheral neuropathy, impaired NMJ function, oxidative stress, and chronic inflammation, exacerbating sarcopenia [62,64]. Vitamin B12 is also utilized by methylmalonyl-CoA mutase, and its deficiency causes methylmalonic acid accumulation and abnormal fatty acid synthesis that is incorporated into myelin, causing demyelination and nerve damage that impairs innervation and promotes weakness [63]. Vitamin B12 deficiency further impairs folate metabolism and DNA synthesis, hinders myocyte proliferation and regeneration, and disturbs energy and protein homeostasis via complex interactions with other B vitamins [64,65]. Consistent with these mechanisms, a 2-year longitudinal study of community-dwelling older Korean adults found that vitamin B12 insufficiency independently predicted incident sarcopenia, providing direct clinical support for the pathways described above [67]. Clinical, epidemiological, and mechanistic evidence collectively suggests an important role of vitamin B12 in neuromuscular function and skeletal muscle health, although the efficacy of supplementation for preventing sarcopenia requires further validation.

### 9. Limitations and future directions

The mechanistic links between vitamin B deficiency and sarcopenia described in this review are primarily derived from cellular and animal studies, highlighting several important limitations.

First, quantitative human evidence is limited. Although mechanisms such as reduced NAD availability, impaired SIRT1 activity, and suppressed mTOR signaling are biologically plausible, dose-response relationships between vitamin B deficiency and clinical outcomes (e.g., grip strength and appendicular skeletal muscle mass index) have rarely been established. Defining clinically relevant thresholds associated with muscle decline, such as serum PLP or homocysteine concentrations, would improve screening strategies.

Second, confounding factors remain a major challenge. Vitamin B deficiencies in older adults often coexist with poor nutrition, malabsorption, chronic kidney disease, and polypharmacy, all of which are independent risk factors for sarcopenia [55]. Consequently, observational studies cannot easily distinguish between causality and shared frailty-related factors. Future studies should incorporate stronger causal inference approaches such as Mendelian randomization.

Third, interventional evidence is limited. Most mechanistic data originated from deficiency models, whereas randomized controlled trials evaluating vitamin B supplementation for muscle strength, mass, or physical function are limited. Although vitamin B12 insufficiency has been associated with incident sarcopenia in older adults [67], this observational evidence does not establish treatment efficacy. Future trials should include participants with confirmed biochemical deficiencies, standardized sarcopenia criteria (e.g., AWGS or EWGSOP2), and objective outcomes, such as grip strength, gait speed, and appendicular muscle mass.

Overall, the current evidence supports the biological plausibility that vitamin B deficiency contributes to sarcopenia. However, translation into clinical recommendations requires robust human studies, improved causal analyses, and well-designed interventional trials.

## Vitamin B deficiency and myokine regulation

In addition to their direct metabolic and inflammatory effects, emerging evidence suggests that deficiencies in several B vitamins influence myokine expression and signaling, thereby affecting muscle homeostasis and inter-organ communication (Fig. 2). Alterations in vitamin B status may shift the balance between anabolic and catabolic myokines, contributing to impaired protein synthesis, enhanced proteolysis, mitochondrial dysfunction, and ultimately, sarcopenia. Although most evidence originates from experimental models, this represents a promising and underexplored mechanistic pathway that links micronutrient deficiency to muscle aging. Accordingly, the proposed interactions among vitamin B deficiency, ER stress/ISR activation, and myokine regulation should be regarded as a hypothesis-generating mechanistic framework rather than as definitive evidence of causality, pending validation in well-designed human studies.

### 1. Myokines in muscle homeostasis and sarcopenia

Skeletal muscles secrete numerous biologically active cytokines and peptides, collectively termed myokines, which are released particularly during muscle contraction [68]. Through autocrine, paracrine, and endocrine pathways, myokines regulate skeletal muscle itself as well as distant organs, including the brain, adipose tissue, liver, and bone, establishing skeletal muscle as an endocrine organ [69,70]. Their secretion is markedly stimulated by exercise, linking muscle contraction to systemic metabolic regulation [71].

Hundreds of myokines have been identified, and their secretion is influenced by exercise, nutrition, and sleep [72,73]. Among them, irisin promotes myogenesis, adipose browning, and cognitive function, and has been proposed as a potential biomarker and therapeutic target for sarcopenia [73,74]. IGF-1 stimulates muscle growth, regeneration, and hypertrophy; IL-15 enhances protein synthesis and reduces adiposity. Conversely, myostatin suppresses muscle hypertrophy and promotes muscle atrophy. IL-6 deserves particular attention because its physiological effects depend on the context; acute exercise-induced IL-6 supports glucose and lipid metabolism, whereas chronically elevated IL-6 contributes to inflammation and muscle catabolism [68,72]. Brain-derived neurotrophic factor also contributes to neuromuscular function via its roles in neurogenesis and cognition [68].

### 2. Effects of vitamin B deficiency on anabolic and catabolic myokines

Evidence directly linking vitamin B deficiency to altered myokine expression remains limited and is predominantly derived from experimental studies. Nevertheless, the available findings suggest that deficiencies in several B vitamins disrupt the balance between anabolic and catabolic myokines.

Experimental evidence suggests that adequate vitamin B6 status contributes to cellular redox homeostasis through antioxidant pathways, including NRF2 signaling. As NRF2 regulates the expression of several exercise-responsive myokines, impaired antioxidant defense during vitamin B6 deficiency may indirectly influence myokine production involved in muscle metabolism, regeneration, and inflammatory responses, although direct evidence remains limited [23,70,72]. Similarly, folate (vitamin B9) deficiency may impair skeletal muscle regeneration through disturbances in one-carbon metabolism, DNA synthesis, oxidative stress, and inflammatory signaling rather than through direct effects on myokine regulation [14,23]. Whether folate deficiency directly alters anabolic or catabolic myokine profiles remains to be established. Similarly, vitamin B12 deficiency may promote a more catabolic muscle environment through hyperhomocysteinemia, chronic inflammation, oxidative stress, and neuromuscular dysfunction, which could secondarily influence myokine signaling and impair muscle regeneration. However, there is limited direct evidence linking vitamin B12 deficiency to specific alterations in circulating anabolic and catabolic myokines [13,67].

Collectively, these observations support a biologically plausible but hypothesis-generating model in which deficiencies in several B vitamins may shift myokine signaling from an anabolic to a catabolic phenotype, thereby contributing to age-related muscle loss.

### 3. Endoplasmic reticulum stress-integrated stress response-mediated regulation of stress myokines

Emerging molecular evidence indicates that deficiencies in riboflavin (vitamin B2) and pyridoxine (vitamin B6) activate ER stress and ISR in skeletal muscles. Activation of the eukaryotic translation initiation factor 2 alpha kinase pathway promotes selective translation of ATF4, a master regulator of cellular stress adaptation. Sustained activation of the ATF4 pathway during chronic vitamin B deficiency is associated with increased expression of the stress-responsive mitokines/myokines GDF15 and FGF21. Although transient elevations in GDF15 and FGF21 facilitate adaptive metabolic responses, persistent activation under chronic nutritional stress appears to exert deleterious effects. Elevated GDF15 levels have been associated with muscle wasting and mitochondrial dysfunction, whereas chronic FGF21 signaling may promote amino acid imbalance and accelerate muscle protein degradation through activation of UPS and autophagy pathways.

Taken together, the ER stress–ISR–ATF4–GDF15/FGF21 axis may represent an important mechanistic link between vitamin B deficiency and age-related sarcopenia. However, because the current evidence is derived primarily from experimental models, future longitudinal cohort studies and randomized controlled trials integrating vitamin B profiling with circulating myokines, GDF15, FGF21, and other stress-response biomarkers are needed to validate this hypothesis-generating framework and determine its clinical relevance and therapeutic potential.

## Clinical implications and future perspectives

### 1. Clinical implications

#### 1) Clinical significance of vitamin B–myokine interactions

Emerging evidence suggests that interactions between vitamin B status and myokine regulation have important clinical implications for the early identification, risk stratification, and monitoring of sarcopenia [14,63]. Alterations in circulating anabolic myokines (e.g., IGF-1, irisin, and IL-15) together with increases in catabolic and inflammatory myokines (e.g., myostatin, IL-6, and TNF-α) may reflect progressive deterioration of muscle homeostasis. However, clinically meaningful cutoff values and standardized reference ranges have not yet been established, and prospective validation is required before these biomarkers can be incorporated into routine practice [66].

Monitoring myokine profiles following nutritional intervention or vitamin B supplementation may provide an objective means of assessing the biological responses to treatment. Likewise, therapies targeting myokine regulation, including the enhancement of muscle-beneficial myokines (e.g., IL-7 and secreted protein acidic and rich in cysteine) through NRF2 modulation or inhibition of the myostatin/activin pathway, may represent promising therapeutic strategies, although their efficacy in vitamin B deficiency-associated sarcopenia remains to be established [63,66,75]. Furthermore, aligning these novel myokine and metabolic biomarkers with consensus-based diagnostic criteria, such as the EWGSOP2 and AWGS 2019 frameworks that define sarcopenia using standardized cutoffs for muscle strength, mass, and physical performance, could strengthen their utility in clinical practice and baseline risk stratification.

Beyond conventional myokines, stress-responsive myokines/mitokines, such as GDF15 and FGF21, may provide complementary biomarkers reflecting activation of the ER stress–ISR pathway induced by vitamin B deficiency. The combined assessment of these biomarkers and inflammatory myokines may improve our understanding of disease activity and therapeutic responses. In addition, chronic inflammatory changes associated with vitamin B12 deficiency may contribute to insulin resistance, cardiovascular risk, and NMJ dysfunction, further supporting the systemic clinical relevance of vitamin B–myokine interactions [64].

#### 2) Homocysteine as a practical surrogate biomarker

Although the direct measurement of individual B vitamins is informative, assays for thiamine, riboflavin, and pyridoxine are often expensive, technically demanding, and not routinely available in many clinical laboratories or long-term care facilities. Consequently, serum homocysteine represents a practical surrogate biomarker reflecting functional deficiencies of folate (vitamin B9), vitamin B12, and vitamin B6 through disruption of the remethylation and transsulfuration pathways.

Elevated homocysteine is not only an indicator of impaired vitamin B metabolism, but is also implicated in mitochondrial oxidative stress, chronic inflammation, endothelial dysfunction, and NMJ impairment, all of which contribute to skeletal muscle deterioration [76]. Cross-sectional and longitudinal studies have demonstrated independent associations between elevated serum homocysteine levels and reduced appendicular skeletal muscle mass, handgrip strength, and gait speed in hospitalized and community-dwelling older adults [10,67]. Given that homocysteine testing is inexpensive, standardized, and widely available in routine laboratory medicine, its incorporation into geriatric assessment may facilitate the identification of older adults who could benefit from further evaluation of their vitamin B status and targeted nutritional interventions. However, its specificity for sarcopenia remains limited because hyperhomocysteinemia may also reflect renal dysfunction, cardiovascular disease, and other comorbid conditions.

#### 3) Integrating exercise and nutrition strategies

Exercise is the most effective physiological stimulus of myokine secretion. Both resistance and aerobic exercise substantially modulate myokine profiles, largely independent of vitamin B status, while improving muscle mass, strength, and metabolic homeostasis [77]. Furthermore, organokine crosstalk between skeletal muscle, adipose tissue, the liver, and other organs contributes to systemic adaptations during physical activity [78]. Accordingly, combined interventions that integrate structured exercise, adequate protein intake, and correction of vitamin B deficiency are likely to provide greater clinical benefits than vitamin B supplementation alone. Currently, no vitamin B-specific pharmacotherapy has been approved for sarcopenia and management of muscle loss continues to rely primarily on multimodal lifestyle interventions.

#### 4) Precision nutrition and personalized management of sarcopenia

Advances in biomarkers, multi-omics, and AI have enabled precision nutrition for sarcopenia. The integration of vitamin B status, homocysteine and myokine profiles, and multi-omics data may improve early risk prediction, support personalized vitamin B supplementation and exercise strategies, and facilitate the monitoring of therapeutic responses. Although further validation is required, these approaches represent a promising step toward personalized prevention and management of sarcopenia.

### 2. Future perspectives

The current evidence linking vitamin B deficiency to sarcopenia is predominantly derived from mechanistic and observational studies, and a causal relationship remains to be established. Large prospective cohort studies and randomized controlled trials are needed to determine the optimal types, dosages, and timing of vitamin B supplementation, and to establish whether correction of vitamin B deficiency can prevent or delay the progression of sarcopenia. These studies should also validate myokine- and homocysteine-based biomarkers using standardized diagnostic criteria and clinically meaningful outcome measures. Although pantothenic acid (vitamin B5) and biotin (vitamin B7) play established roles in CoA-dependent energy metabolism, fatty acid oxidation, and carboxylation reactions, direct evidence linking deficiencies of these vitamins to sarcopenia remains scarce. Further mechanistic and clinical studies are required to clarify their contributions to skeletal muscle homeostasis.

Future investigations should further elucidate how vitamin B deficiency affects ER stress, ISR, ATF4 signaling, and downstream stress-responsive myokines/mitokines, including GDF15 and FGF21. Clarifying these pathways may help identify novel therapeutic targets and deepen our mechanistic understanding of muscle aging. In parallel, the integration of vitamin B profiling, homocysteine levels, myokine levels, and ER stress biomarkers with multi-omics data and AI-assisted prediction models may enable precision nutrition, personalized vitamin B supplementation and exercise interventions, and objective monitoring of therapeutic responses, ultimately supporting the individualized prevention and management of sarcopenia.

## Conclusion

Sarcopenia is a multifactorial age-related disorder in which nutritional status is an important and potentially modifiable determinant of muscle health. Current evidence suggests that vitamin B deficiency contributes to skeletal muscle dysfunction via interconnected mechanisms involving impaired mitochondrial energy metabolism, oxidative stress, chronic inflammation, neuromuscular dysfunction, dysregulated protein turnover, ER stress/ISR activation, and altered myokine signaling. Although the strength of evidence varies among individual vitamin B subtypes, many mechanistic observations remain derived from experimental studies and these findings support a biologically plausible, hypothesis-generating framework rather than a definitive causal model. Future longitudinal cohort studies and randomized controlled trials are required to validate these mechanisms and determine whether vitamin B-related biomarkers can improve risk stratification and guide precision nutrition strategies for the prevention and management of sarcopenia. This review proposes an integrated mechanistic framework that links vitamin B deficiency, ER stress/ISR activation, and myokine dysregulation as converging pathways that may accelerate sarcopenia.

## Figures and Tables

**Fig. 1.**
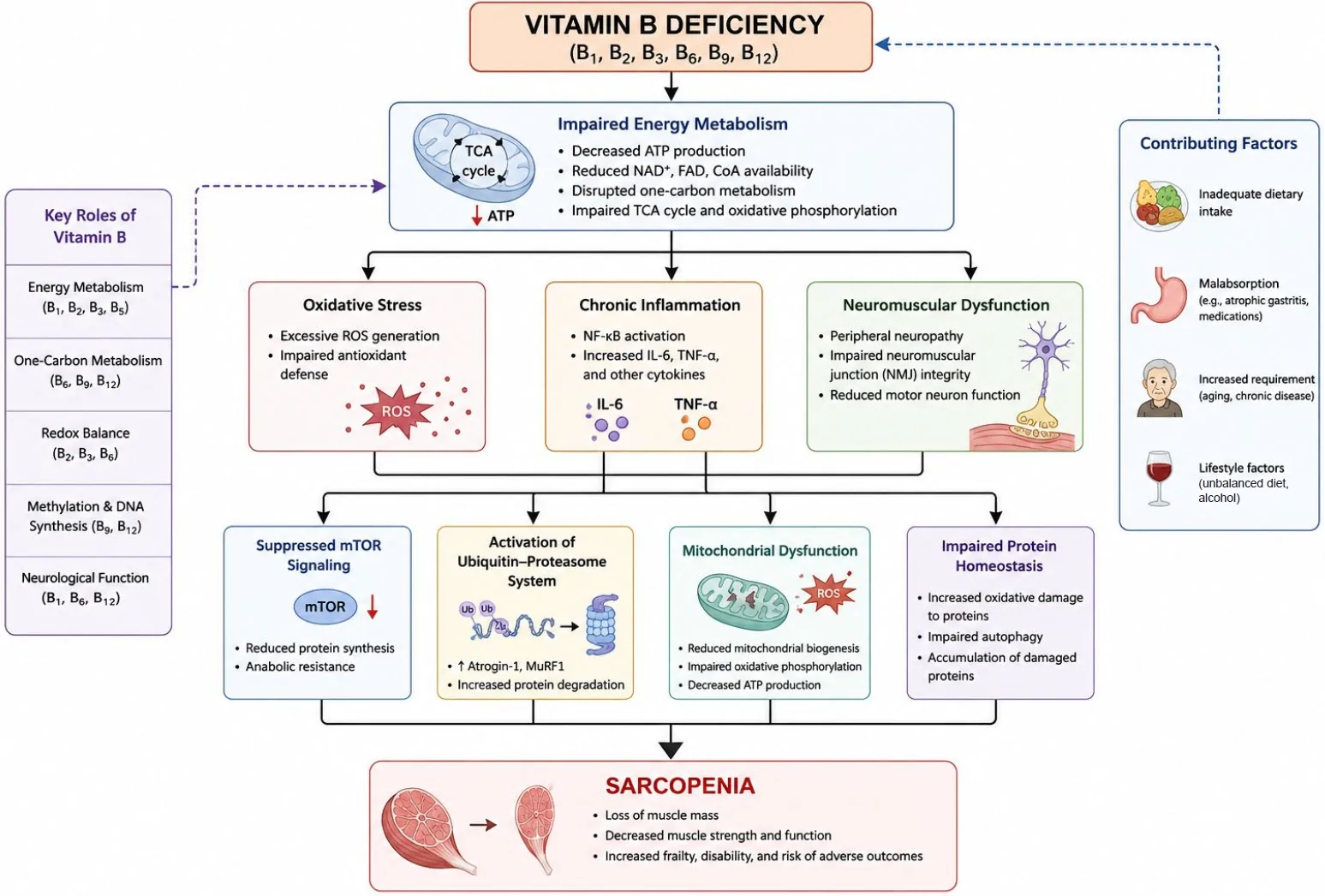
Schematic representation of the pathophysiological axis linking vitamin B deficiency to sarcopenia. Vitamin B deficiency impairs energy metabolism by reducing ATP production and cofactor availability, leading to oxidative stress, chronic inflammation, and neuromuscular dysfunction. These alterations suppress mTOR signaling, activate the ubiquitin-proteasome system (UPS), disrupt mitochondrial function, and impair protein homeostasis, ultimately promoting sarcopenia. The strength of evidence differs substantially among vitamin B subtypes. Associations involving vitamins B6, B9, and B12 are supported by relatively consistent epidemiological studies, clinical observations, and mechanistic investigations, whereas evidence for vitamins B1, B2, B3, and B5 relies predominantly on experimental and preclinical studies. For vitamin B7, direct evidence linking deficiency to sarcopenia remains scarce, and proposed mechanisms are largely speculative. Accordingly, the mechanistic pathways summarized in this review should be interpreted in the context of their respective levels of evidence. ATP, adenosine triphosphate; NAD+, nicotinamide adenine dinucleotide; FAD, flavin adenine dinucleotide; CoA, coenzyme A; TCA, tricarboxylic acid cycle; ROS, reactive oxygen species; NF-κB, nuclear factor kappa B; IL-6, interleukin-6; TNF-α, tumor necrosis factor alpha; NMJ, neuromuscular junction; mTOR, mechanistic target of rapamycin; MuRF1, muscle RING-finger protein-1. The figure has been created by the authors with assistance from ChatGPT (OpenAI). The authors have reviewed and verified the final figure for scientific accuracy.

**Fig. 2.**
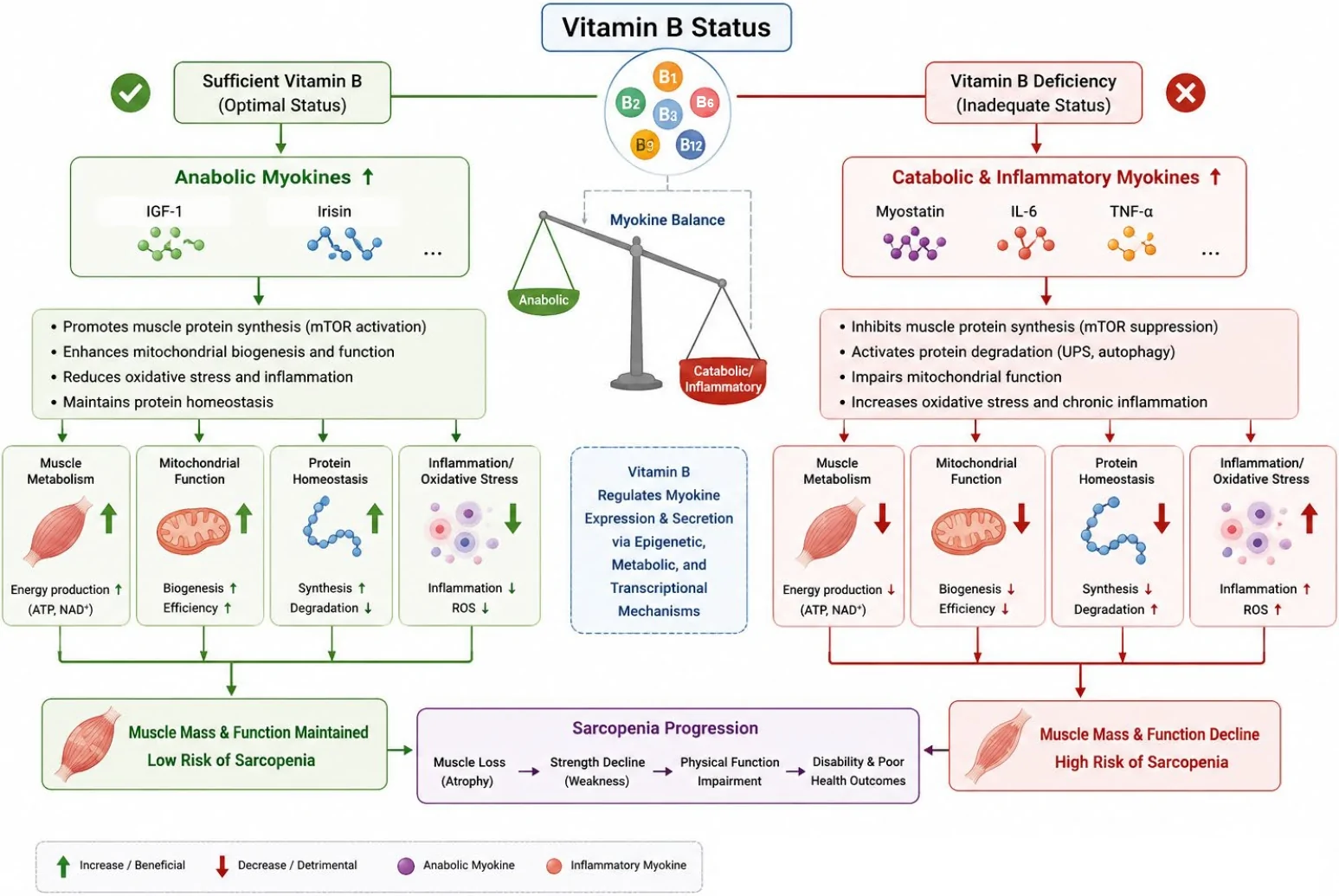
Integrated mechanistic framework linking vitamin B status to myokine regulation and sarcopenia. Vitamin B deficiency disrupts mitochondrial energy metabolism, redox homeostasis, one-carbon metabolism, and neuromuscular function, triggering endoplasmic reticulum stress and activation of the integrated stress response (ISR). Persistent ISR signaling induces activating transcription factor 4, leading to increased expression of stress-responsive mitokines/myokines, including growth differentiation factor 15 and fibroblast growth factor 21. These signaling cascades shift the myokine balance toward catabolic and inflammatory mediators (e.g., myostatin, activin A, interleukin [IL]-6, and tumor necrosis factor alpha [TNF-α]) over anabolic myokines (e.g., insulin-like growth factor-1 [IGF-1], irisin, and IL-15). This imbalance suppresses mechanistic target of rapamycin (mTOR)-mediated protein synthesis, accelerates proteolysis via the ubiquitin-proteasome system (UPS) and autophagy, and promotes mitochondrial dysfunction, ultimately driving muscle atrophy and age-related sarcopenia progression. The figure was created by the authors using ChatGPT (OpenAI) and finalized after critical review for scientific accuracy. ATP, adenosine triphosphate; NAD⁺, nicotinamide adenine dinucleotide; ROS, reactive oxygen species. The figure has been cre­ated by the authors with assistance from ChatGPT (OpenAI). The authors have reviewed and verified the final figure for scientific accuracy.

**Table 1.** Mechanistic links between vitamin B deficiency and sarcopenia

Vitamin	Primary physiological role	Key mechanisms of deficiency	Major molecular pathways affected	Consequences for muscle
Vitamin B1 (thiamine)	Carbohydrate metabolism, ATP production	Reduced activity of PDC and α-KGDH → ATP depletion, ROS accumulation	mTOR ↓, mitochondrial dysfunction, NMJ impairment	Muscle weakness, neurogenic atrophy
Vitamin B2 (riboflavin)	Redox reactions (FAD, FMN), ETC function	Impaired ETC, decreased glutathione regeneration → oxidative stress	ETC dysfunction, antioxidant defense ↓, ER stress (UPR activation)	Muscle cell damage, atrophy
Vitamin B3 (niacin)	NAD+/NADP+ synthesis, energy metabolism	Reduced NAD+ levels → impaired OXPHOS, increased ROS	SIRT1 ↓, PGC-1α ↓, UPS ↑	Mitochondrial dysfunction, protein degradation
Vitamin B5 (pantothenic acid)	Coenzyme A synthesis, TCA cycle	Impaired acetyl-CoA production → reduced ATP and fatty acid oxidation	mTORC1 ↓, metabolic stress pathways	Reduced protein synthesis, muscle atrophy
Vitamin B6 (pyridoxine)	Amino acid metabolism, coenzyme (PLP)	Impaired amino acid utilization, increased inflammation, hyperhomocysteinemia	AKT/mTOR ↓, NF-κB ↑, NLRP3 inflammasome activation	Enhanced protein breakdown, impaired regeneration
Vitamin B7 (biotin)	Carboxylation reactions, energy metabolism	Impaired pyruvate carboxylase and leucine metabolism → ATP depletion	AMPK ↑, mTORC1 ↓	Reduced protein synthesis, increased catabolism
Vitamin B9 (folate)	One-carbon metabolism, DNA synthesis	Impaired DNA repair and methylation, hyperhomocysteinemia	AKT/mTOR ↓, DDIT4 ↑	Impaired muscle regeneration, increased apoptosis
Vitamin B12 (cobalamin)	Methionine cycle, nerve function	Homocysteine accumulation, methylmalonic acid increase → neuropathy	NMJ dysfunction, oxidative stress, inflammation	Muscle weakness, neurogenic atrophy

ATP, adenosine triphosphate; PDC, pyruvate dehydrogenase complex; α-KGDH, α-ketoglutarate dehydrogenase; ROS, reactive oxygen species; mTOR, mechanistic target of rapamycin; NMJ, neuromuscular junction; FAD, flavin adenine dinucleotide; FMN, flavin mononucleotide; ETC, electron transport chain; ER, endoplasmic reticulum; UPR, unfolded protein response; NAD+, nicotinamide adenine dinucleotide; NADP+, nicotinamide adenine dinucleotide phosphate; OXPHOS, oxidative phosphorylation; SIRT1, sirtuin 1; PGC-1α, peroxisome proliferator-activated receptor gamma coactivator 1-alpha; UPS, ubiquitin-proteasome system; CoA, coenzyme A; TCA cycle, tricarboxylic acid cycle; PLP, pyridoxal 5′-phosphate; NF-κB, nuclear factor kappa B; NLRP3, NOD-like receptor family pyrin domain containing 3; AMPK, AMP-activated protein kinase; DDIT4, DNA damage-inducible transcript 4.

**Table 2.** Integrated pathophysiological mechanisms linking vitamin B deficiency to sarcopenia

Pathophysiological mechanism	Effects of vitamin B deficiency	Impact on sarcopenia progression
Mitochondrial dysfunction and impaired energy metabolism	Reduced ATP production, impaired NAD+ and CoA availability, disrupted oxidative phosphorylation	Reduced muscle strength, endurance, and increased fatigue
Oxidative stress	Excessive ROS generation and impaired antioxidant defense	Oxidative damage to proteins, lipids, mitochondria, and DNA
Chronic inflammation	NF-κB activation and increased IL-6, TNF-α, and other inflammatory mediators	Accelerated muscle protein degradation and anabolic resistance
Neuromuscular dysfunction	Peripheral neuropathy, impaired neuromuscular junction integrity, reduced motor neuron function	Decreased muscle activation, coordination, and physical performance
Protein turnover imbalance	Suppressed mTOR signaling, activation of the ubiquitin-proteasome and autophagy pathways	Net muscle protein loss and muscle atrophy
Impaired muscle regeneration	Satellite cell dysfunction, impaired DNA synthesis, and reduced regenerative capacity	Delayed muscle repair and recovery
Myokine dysregulation	Altered IGF-1, myostatin, irisin, IL-6, FGF21, and GDF15 signaling	Disrupted muscle homeostasis and progression of sarcopenia

ATP, adenosine triphosphate; NAD+, nicotinamide adenine dinucleotide; CoA, coenzyme A; ROS, reactive oxygen species; DNA, deoxyribonucleic acid; NF-κB, nuclear factor kappa B; IL-6, interleukin-6; TNF-α, tumor necrosis factor alpha; mTOR, mechanistic target of rapamycin; IGF-1, insulin-like growth factor-1; FGF21, fibroblast growth factor 21; GDF15, growth differentiation factor 15.
